# Research on evaluation index method of cloud-network convergence capability

**DOI:** 10.1038/s41598-023-47626-3

**Published:** 2023-11-20

**Authors:** Tengteng Ma, Yuanmou Chen, Haobin Wang, Xiaoming Xiong, Jing Zhao

**Affiliations:** Strategy Development Institute, China Telecom Corporation Limited Beijing Research Institute, Beijing, 102209 China

**Keywords:** Electrical and electronic engineering, Information technology

## Abstract

There is no measurable and evaluable index system for cloud-network convergence that provides guidance and reference for the subsequent construction and development of cloud-network convergence. It is a big project to select and evaluate the indexes of cloud-network convergence, which requires suitable index selection and index evaluation schemes. Based on analytic hierarchy process (AHP) and entropy weight method, this paper proposes an improved AHP (i-AHP) index selection scheme and index evaluation scheme leveraging the years of experts’ experience, the geometric mean and the least square method. The improved weighted least square method (WLSM) is finally proved to be more stable for index evaluation scheme by adding abnormal data. In addition, the index weight obtained by the index evaluation scheme with WLSM are provided as a reference for the future development of cloud-network convergence. The simulation results show that the proposed scheme is superior to the existing index evaluation scheme and can avoid the weight deviation caused by the disturbance and fluctuation of abnormal data.

## Introduction

**Figure 1:**
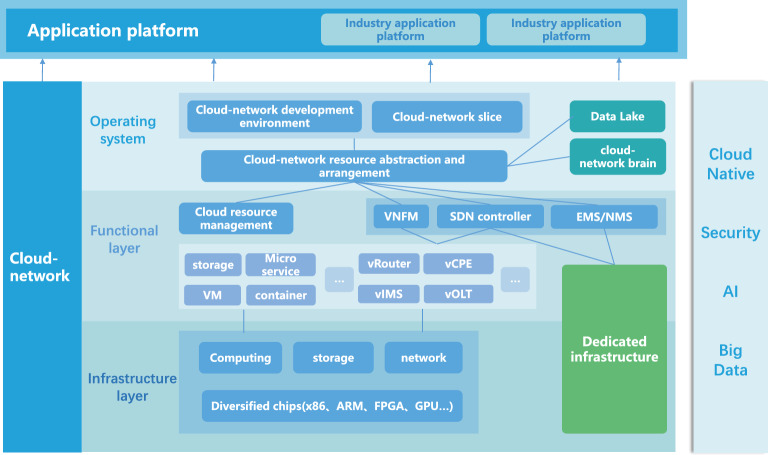
China telecom cloud-network convergence target technology architecture^1^.

With the introduction of national strategies such as “new infrastructure” and “digital economy”, cloud-network convergence has become an inevitable trend in the evolution and upgrading of 5G/6G, cloud computing and artificial intelligence (AI). In addition, it is significant to solve the problem of cloud and network reconfiguration to meet the demands of diversified traffic. Figure 1 shows the target technology architecture of cloud-network convergence proposed by China Telecom. Cloud-network convergence is a profound change of information infrastructure by the integration of communication and information technologies. There are three stages, i.e., coordination, convergence, and integration for the development of cloud-network convergence. Finally, the independent cloud computing resources and network facilities are merged to form a system of integrated supply, integrated operation and integrated service^1^. The research of cloud-network convergence is of great significance to promoting the digital and intelligent upgrading of industries, such as telecommunication^2,3^, power grid^4^, industrial Internet^5^, Internet of Things (IoT)^6–8^, and medical^9^.

The innovation and upgrading of basic technical elements such as network, cloud, AI, security, green and related applications are emerging, which makes the development of cloud-network convergence constantly updated and iterated. The development level of cloud-network convergence reflects the digitalization and intelligence of infrastructure for telecom operators, and its construction level is closely related to the operators’ strategic development. In order to estimate the development and construction level of cloud-network convergence capability, it is urgent to build a comprehensive and effective evaluation system for cloud-network convergence capability.

However, the evaluation of existing cloud and network pays more attention to a single resource or characteristic capability. For example, for the evaluation of cloud resources^10^, proposed a cloud resource evaluation model, which is called entropy optimization evaluation and ant colony clustering model (EOEACCM). Regarding network security evaluation, the network security on-site evaluation was established, and the network evaluation objects and evaluation methods were accurately selected based on different user access paths in^11^. In order to overcome the network security problems in the current smart city construction process, a novel smart city network security evaluation system was put forward^12^. The above cloud and network evaluation are difficult to meet the new requirements of the existing cloud-network convergence development.

In addition, the evaluation of cloud-network convergence is complicated, which is mainly reflected in two aspects. One is that for cloudization of the network by network functions virtualization (NFV) and the network elements on the cloud platform, it is difficult to reflect the overall service capability of the network simply by evaluating the network elements’ capabilities. We need to comprehensively evaluate the overall capabilities of the cloud and network elements, such as network interface card, single root I/O virtualization (SR-IOV), data plane development kit (DPDK), CPU load and scheduling, network element management and arrangement, network slicing/tunneling, forwarding, and routing, etc. The other is that cloud-network convergence reflects the end-to-end service capability, that is, cloud-network as a service (CNaaS). When users’ quality of service (QoS) drops rapidly, it is necessary to position the shortcomings and congestion points of cloud-network by cloud-network convergence index evaluation. Thus, how to select key indexes and how to effectively balance the accuracy of index evaluation is a significant problem in the cloud-network convergence evaluation system.

In the thesis, we design an evaluation system of cloud-network convergence to select the indexes and demine the weight of each index, where convergence capability score is used to evaluate convergence capability by convergence indexes’ scores and convergence indexes’ weights. The main contributions of this article are summarized as follows:

- Based on AHP, this paper proposes an improved AHP (i-AHP) index selection scheme that can deal with the abnormal index dimension caused by expert misjudgment and get the top *N* indexes for the evaluation of cloud-network convergence index.
- We set the years of experts’ experience as the weight value of the geometric mean and the least square method, and combine AHP and EWM to design the comprehensive index weight schemes.
- Multiple schemes for the comprehensive index weight are tested by adding abnormal data. Finally, compared with other schemes, the weighted least square method (WLSM) is determined as the most stable method.

The rest of this paper is organized as follows. Section “Related work” introduces the related work to this paper. In Section “System model and solution schedule”, the evaluation system model and cloud-network convergence evaluation algorithm are described. The simulation results are shown in Section “Simulation results” and followed by the conclusions in Section “Conclusion”.

## Related work

The establishment of the evaluation system is generally divided into three parts, namely, selecting evaluation indexes, determining the weight of indexes, and comprehensively scoring^13^. There are two kinds of methods for selecting evaluation indexes, including qualitative analysis and quantitative analysis. However, the single qualitative analysis is highly subjective such as the expert consultation method^14^. Single quantitative analysis, such as principal component analysis^15^, requires data with high correlation, but the existing data is hard to meet the data quality requirements. Therefore, the combination of qualitative and quantitative methods is adopted as an objective index selection scheme, which reduces the difficulty of data acquisition.

Recently, there are two main categories of methods for determining weights, qualitative analysis including analytic hierarchy process (AHP)^16–19^ and quantitative analysis including fuzzy cluster analysis^20^, entropy weight method (EWM)^21–23^, and index weight determination method based on principal component analysis^24^, etc. Fuzzy clustering is suitable for clustering large-scale data by calculating the uncertainty degree of samples belonging to various categories and performing clustering analysis. Principal component analysis (PCA) is a statistical method for dimensionality reduction, which transforms the original random vector whose components are related into a new random vector whose components are irrelevant, and is suitable for the evaluation with strong correlation of multiple indexes. Our evaluation index data of cloud-network convergence capability is small in scale, and the correlation between indexes is low, which is more suitable for AHP and EWM. The AHP and EWM are two most commonly used evaluation methods. The AHP method adopts the subjective data of index pairwise comparison as the index evaluation standard to obtain the index weight. The AHP was utilized to construct the evaluation index system of sports tourism suitability based on the comprehensive consideration of different factors such as resource conditions, environmental conditions, and location, providing a foundation for sustainable tourism development in^17^. The integrated Structure-Actor-Water framework (iSAW) framework and AHP method were used to identify the indicators preferred by stakeholders to choose the best sustainable irrigation system in Ardabil Province, Iran, indicating that all groups prefer pressurized irrigation systems with employment and income being the most important indicators for decision-making in^19^. EWM is an objective weighting method and the weight is obtained based on the dispersion degree of each index data^21^. Summarized the status of combined evaluation models using the EWM, including its concepts, models, types and existing models.^22^ presented a LED harmonic evaluation approach using the G1 and entropy methods to calculate weights of harmonic characteristics and thoroughly represent harmonic emissions, aiming to improve power quality of distribution network. In^23^, this bibliometric study provides a comprehensive analysis of the utilization and impact of EWM in machining operations.

However, as the development and construction of cloud-network convergence involve many fields, such as cloud, network, data, service, security, green, and so on, the dimension design of its evaluation index is complicated. The use of a single AHP or EWM would lead to some problems, such as non-objective and unstable weight value. In order to avoid the subjective judgement of a single expert and the clerical error in scoring, it is necessary to give the final evaluation result by the combination of AHP and EWM, which realizes both objective and common sense judgement of weight assignment^25^. The combination of AHP and EWM has been studied in^16^ and a comprehensive utility evaluation model was proposed, through AHP to evaluate performance and value, entropy methods to evaluate objective attributes, and combining subjective and objective weights to obtain a final comprehensive utility evaluation weight. In^26^, a combination of subjective and objective weight design scheme based on EMW and AHP was put forward, and the combined weight was optimized by Lagrange multiplier method for the evaluation of body modeling design. In^27^, based on AHP and improved EWM, a comprehensive evaluation method combining subjective and objective was proposed to evaluate the equipment utilization rate of a medium voltage distribution network in Guangdong.^28^ put forward a weight design scheme that first calculates the weight with AHP, and then modified the weight with EWM to evaluate urban comprehensive transportation network.

Different from the^16,26–28^ that made a comprehensive evaluation weight from the perspective of weighted summation and alternate revision of weight design scheme, in the paper, an evaluation scheme is designed, which combines expert experience as weighting factors, the weighted geometric mean (WGMM) method, and the WLSM to make the evaluation index weight more stable and accurate. In addition, in view of the fact that some dimension indexes may be wrongly marked by multiple experts, this paper designs a scheme to deal with abnormal dimension indexes, which increases the usability of small sample data by automatically correcting abnormal data.

## System model and solution schedule

**Figure 2:**
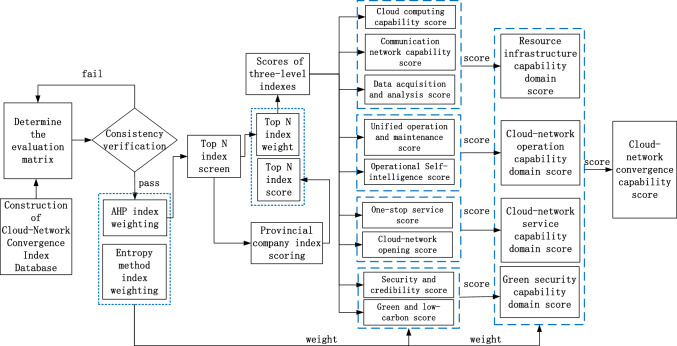
Evaluation system process of cloud-network convergence.

The establishment of a cloud-network convergence evaluation system includes index construction, index weighting, and index scoring. Index construction refers to the collection of cloud-network convergence experts’ index opinions according to the current situation of cloud-network convergence development in China Telecom, forming the original index set. Index weighting refers to calculating the importance of each index in the original index set by using the proposed algorithm. Index scoring evaluates the cloud-network convergence capability based on the optimized index set and weight. The overall process of the evaluation system of cloud-network convergence in this paper is shown in Fig. 2.

The most significant process in the evaluation is to select and weight indexes. This thesis mainly focuses on the research of index selection method in index construction and index weighting method. We designed a two-step implementation scheme. The first step is index selection. The judgement matrices of indexes are obtained by the scores of many cloud-network convergence experts in the industry, and we propose an i-AHP index selection scheme to weight indexes. According to the weight value, the index corresponding to top *N* is selected as the evaluation index, so as to facilitate the subsequent evaluation and scoring of provincial companies for the cloud-network convergence degree. Secondly, based on the first step, combining with EWM, a group of weight vectors are given. Multiple groups of weight vectors are selected to process this group of weight vectors by WGMM and WLSM in Scheme 2. The weights of the two methods are set according to the experience years of cloud-network convergence experts.

### Scheme 1: i-AHP index selection scheme

This article proposes a new index selection scheme leveraging evaluation matrix of AHP and WGMM, i.e., i-AHP index selection scheme. Firstly, an evaluation matrix template is designed based on the index database, and multiple cloud-network convergence experts in the industry provide evaluation matrices by the template. Then, the consistency of these matrices is verified, and the maximum eigenvalues and eigenvectors of the evaluation matrices that meet the conditions are obtained. Finally, the eigenvectors given by multiple evaluation matrices are averaged using the geometric averaging method to obtain weight values. Based on actual requirements, the top *N* index components in the weight vector ranking are selected, resulting in cloud-network convergence indexes that can be used for subsequent key evaluations.

**Table 1:** Judgement matrix template.

	Index 1	Index 2	...	Index *n*
Index 1	$a_{11}$	$a_{12}$	...	$a_{1n}$
Index 2	$a_{21}$	$a_{22}$	...	$a_{2n}$
$\vdots$	$\vdots$	$\vdots$	$\ddots$	$\vdots$
Index *n*	$a_{n1}$	$a_{n2}$	...	$a_{nn}$

Remark: The values in the matrix are the importance of the corresponding vertical indexes versus the horizontal indexes. For example, $a_{ij}=\frac{n}{m}$ is the importance ratio of the ith index compared with the jth index regarding cloud-network convergence, where $n,m\in (0,9]\cap \mathbb {Z}$. These values ranging from 1 to 9 indicate the importance degree of these indexes for cloud-network convergence, and the greater the value, the greater the importance degree

The main steps of the i-AHP index selection scheme are as follows:

1. Leveraging the indexes of the index database, the judgement matrix template is set in Table 1 and the corresponding judgement matrix is given as follows.
  1 $$\begin{aligned} A={ \left[ \begin{array}{cccc} a_{11} &{}\quad a_{12} &{}\quad \dots &{}\quad a_{1n}\\ a_{21} &{}\quad a_{22} &{}\quad \dots &{}\quad a_{2n}\\ \vdots &{}\quad \vdots &{}\quad \ddots &{}\quad \vdots \\ a_{n1} &{}\quad a_{n2} &{}\quad \dots &{}\quad a_{nn} \end{array} \right] } \end{aligned}$$
2. *m* experts in cloud-network convergence scored the n indexes according to the rules in step 1, and *m* corresponding $n\times n$ order judgement matrices are obtained. We recorded these evaluation matrices as $A_1, A_2, A_3, \dots , A_m$ respectively.
3. Calculate the maximum eigenvalue $\lambda _i$ of the judgement matrix $A_i,i\in [1,m]\cap \mathbb {Z}$ and the corresponding eigenvector $\omega _i$.
4. Through the formula
  2 $$\begin{aligned} CR=\frac{{\lambda _i}-n}{(n-1)RI}, \end{aligned}$$
  the consistency of the judgement matrix is verified. If $CR<0.1$, it is stored in the available database $D_u$; If not, it will be stored in the pending database $D_t$. Calculate every value $a_{ij}$ of these matrices in the available database $D_u$ by geometric mean method, and get the matrix with mean data.
5. Make single data correction to these matrics stored in the pending database $D_t$. Starting from the first index score of the matrics, replace it with the corresponding value of the matrix mean, and carry out consistent verification. If it passes, store the revised matrix in the available database; if it fails, restore the first index score, change the second index score to the matrix mean, and carry out consistent verification. And so on, until the last index still fails to pass the consistent certification, then delete the data.
6. Calculate the eigenvector of the matrix in the database $D_u$. These eigenvector values $\omega _i$ are carried out by the geometric mean method to obtain a reference comprehensive weight vector for index selection.
7. According to the actual requirements, select the top N indexes in weight vector components ranking, that is, the selected important evaluation index.

#### Remark 1

For the specific process of consistency verification regarding the (2), please refer to the literature^29^.

**Table 2:** judgement matrix template.

Weight									
Indexes	Index1: Cloud-computing capability	Index2: Communication network capability	Index3: Data acquisition and analysis	Index4: Unified operation and maintenance	Index5: Operational self-intelligence	Index6: One-stop service	Index7: Cloud-network opening	Index8: Security and credibility	Index9: Green and low-carbon
$w_1$	0.2049	0.2516	0.1227	0.0818	0.0697	0.0661	0.0737	0.0937	0.0358
$w_2$	0.2399	0.2537	0.0549	0.0929	0.0206	0.0809	0.0815	0.1095	0.0661
$w_3$	0.1408	0.1408	0.1149	0.0855	0.0836	0.1408	0.1118	0.1105	0.0714
$w_4$	0.0967	0.0967	0.0967	0.1323	0.1143	0.1143	0.1336	0.1336	0.0818
$w_5$	0.1140	0.1894	0.0936	0.1601	0.0831	0.1140	0.0851	0.0691	0.0915
$w_{\text {iahp}}$	0.1526	0.1598	0.0987	0.1130	0.0769	0.1014	0.1100	0.1140	0.0887
$w_{\text {ewm}}$	0.1174	0.0718	0.0937	0.1072	0.1283	0.0787	0.1649	0.1493	0.073526

### Scheme 2: Index weight design

Existing evaluation index weight methods mainly include AHP and EWM. AHP is a commonly used method to calculate the weight of evaluation indexes. According to the subjective experience, experts make pairwise comparisons and judgements to get judgement matrices, and the index weight vector is given based on the eigenvector of the matrix, which may lead to the misjudgment of experts. Although the EWM objectively weights the indexes according to the variation degree of the index data, which avoids subjective misjudgment of experts, it depends on the actual data of the indexes.

In order to solve the problem that the misjudgment of experts and the unobtainable available index data leads to the unreasonable design of index weights, a new scheme is put forward. The main idea is the way of subjective and objective comprehensive weighting. Firstly, the weight vectors corresponding to multiple experts are obtained by scheme 1, then experts in different fields provide data of these indexes as multiple samples, which is used in EWM to calculate relative objective weight. Finally, combined with the experience years of cloud-network convergence experts, the WGMM and WLSM are set to average and fit these weight vectors. The main steps of the scheme are as follows.

1. The available database $D_u$ of scheme 1 is executed to determine the N indexes to be evaluated and a set of weight vectors corresponding to the selected evaluation indexes is represented as follows.
  3 $$\begin{aligned} \varvec{\omega ^1}&=\left[ \omega _1^1,\omega _1^1,\ldots ,\omega _N^1\right] \nonumber \\ \varvec{\omega ^2}&=\left[ \omega _1^2,\omega _2^2,\ldots ,\omega _N^2\right] \nonumber \\&\dots \nonumber \\ \varvec{\omega ^m}&=\left[ \omega _1^m,\omega _2^m,\ldots ,\omega _N^m\right] . \end{aligned}$$
2. Score the N indexes, that is, each index is scored by *k* experts involved in related specialties with reference to the actual deployment and operation of the specialty, and a matrix of $k\times N$ order is obtained.
3. The EWM^30^ is applied to process and calculate the matrix of $k\times N$ order, and the weight vector
  4 $$\begin{aligned} \varvec{v}&=\left[ v_1, v_2,\ldots , v_N\right] \end{aligned}$$
  corresponding to the indexes is obtained.
4. For the *m* weight vectors obtained in step 1 and the weight vectors obtained in step 3, we leverage cloud-network convergence expert’s experience years $\beta _j$ as the weight of experts’ corresponding index scoring in the algorithm. And the WGMM and WLSM are adopted to get more reasonable index weight vectors as follows
  5 $$\begin{aligned} \varvec{\omega ^{opt1}}=\left[ \omega _1^{opt1},\omega _1^{opt1},\ldots ,\omega _N^{opt1}\right] , \end{aligned}$$
  and
  6 $$\begin{aligned} \varvec{\omega ^{opt2}}=\left[ \omega _1^{opt2},\omega _2^{opt2},\ldots ,\omega _N^{opt2}\right] . \end{aligned}$$

The models and solutions of the two methods in scheme 2 are as follows:

#### Weighted geometric mean

The formula is obtained by WGMM to solve the weight of the final index *i*.

7 $$\begin{aligned} \omega _i^{opt1}=\root \sum \limits _{j=1}^{m+1} \beta _j \of {\left( \prod \limits _{j=1}^m(\omega _i^j)^{\beta _j}\right) \cdot (v_i)^{\beta _{m+1}}}, \end{aligned}$$

where $\beta _j\in \mathbb {Z^+}$ is years of experts’ experience.

#### Weighted least square method

The WLSM is our final choice, and the optimal index weight vector $\varvec{\omega ^{opt2}}$ is fitted according to the weight vector set obtained in algorithm 2. Different from the calculation method in 3.2, each component $\omega _i^{opt2}$ of $\varvec{\omega ^{opt2}}$ is a variable and needs to be less than 1. Therefore, a constrained nonlinear programming problem is formed to find the optimal weight vector $\varvec{\omega ^{opt2}}$.

The goal of constrained nonlinear programming problem is

8 $$\begin{aligned} \sum \limits _{i=1}^N\left( \sum \limits _{j=1}^m(x_i-\omega _i^j)^2\cdot \beta _j+(x_i-v_i)^2\cdot \beta _{m+1}\right) \end{aligned}$$

and meet the condition

9 $$\begin{aligned} \sum \limits _{i=1}^nx_i=1,x_i > 0. \end{aligned}$$

    We solve the following programming problem to obtain the optimal weight vector, that is, the fitted optimal weight vector.

9a $$\begin{aligned}{} & {} \sum \limits _{i=1}^N\left( \sum \limits _{j=1}^m(x_i-\omega _i^j)^2\cdot \beta _j+(x_i-v_i)^2\cdot \beta _{m+1}\right) \end{aligned}$$

9b $$\begin{aligned}{} & {} st:\nonumber \\{} & {} C1:\sum \limits _{i=1}^Nx_i=1,x_i > 0 \end{aligned}$$

    Since the objective function (9a) is convex function and the equality constraint function (9b) is an affine function, the optimization problem () is a convex optimization problem with an optimal solution^31^. The optimal solution $\omega _i^{opt2}$ can be obtained by Matlab convex optimization package, i.e., the weight of index *i* regarding the corresponding cloud-network convergence capability.

**Table 3:** Simulation parameters.

Parameter	Value
The number of cloud-network convergence experts *m*	5
Experts involved in related specialties *k*	7
The number of selected indexes *N*	9
Random consistency index *RI*^29^	1.46
The experience years of experts $\beta _j,j=1,2,3,4,5$	$\beta _1=3,\beta _2=2,$ $\beta _3/\beta _4/\beta _5=1$

## Simulation results

The purpose of this experiment is to verify the effectiveness of the proposed scheme. Since scheme 1 is nested in scheme 2, The experimental simulations are all verified in Scheme 2. Therefore, assuming that the indexes of top *N* in scheme 1 have been obtained, we will directly analyse the scheme 2.

Taking the cloud-network convergence capability of a provincial company of China Telecom as the goal, this thesis evaluates it from the perspectives of resource infrastructure, cloud-network operation, cloud-network service, and green security. According to the rules in scheme 1, experts in the cloud-network convergence industry scored the selected indexes to obtain multiple sets of evaluation matrices. In addition, based on the steps $$2)--4)$$ in scheme 1, cloud-network convergence capability weight vectors $$w_1, w_2, w_3, w_4, w_5$$ and improved AHP (i-AHP) weight vector $$w_\text {iahp}$$ were obtained, see Table 2. In addition, regarding the EWM, the matrix of $$N\times 7$$ order is obtained by that seven experts from relevant specialties of provincial companies score the selected *N* indexes. The weight vector of the evaluation index is given by the EWM in Scheme 2, see $$w_\text {ewm}$$ in Table 2.

**Figure 3:**
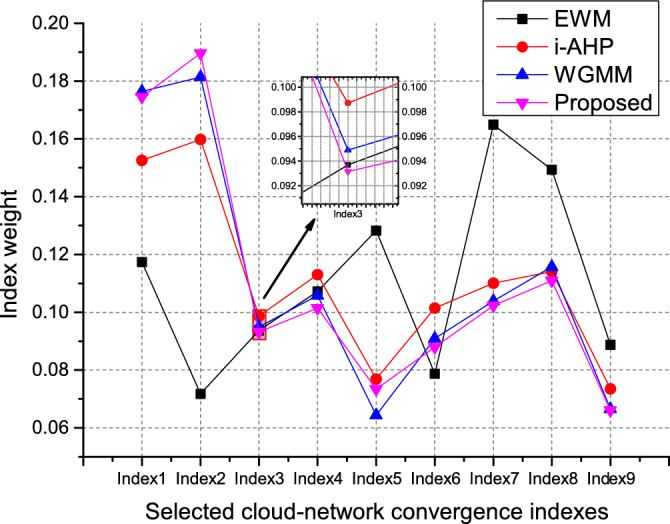
Index weight values corresponding to different algorithms.

Scheme 2 is adopted to deal with the above weight vectors, and the value of the $\beta _j$ parameter is designed according to the years that these experts are familiar with cloud-network convergence. In this simulation, we set the value of $\beta _j$ as $\beta _1=3,\beta _2=2,\beta _3/\beta _4/\beta _5=1$, see Table 3 for other parameters. The improved comprehensive weight value is obtained by WGMM and WLSM, and the cloud-network convergence capability index versus the weight is shown in Fig. 3.

**Figure 4:**
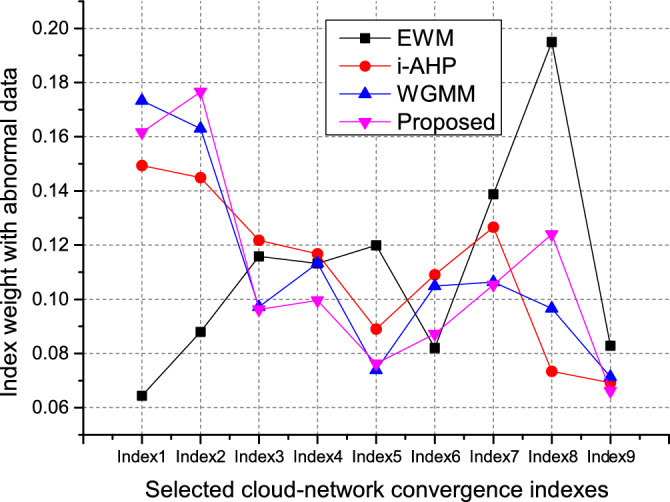
Index weight values corresponding to different algorithms for adding abnormal data.

**Figure 5:**
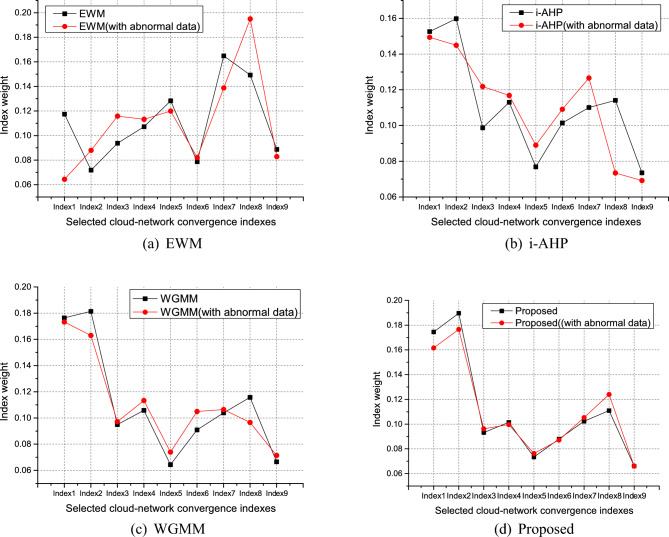
Weight disturbance of different algorithms when adding abnormal data.

In order to verify the effectiveness of the proposed scheme, we added two groups of abnormal data for EWM, i-AHP, WGMM and the proposed scheme, and mixed them with the normal expert scoring data, where abnormal data is randomly generated under the premise that matlab meets the scoring range. Since our method added the years of expert experience as the trade-off parameter of the proposed method, it has stronger stability against the invasion of foreign abnormal data. From Fig. 4, we can see the weights of the proposed scheme, the WGMM, the i-AHP, and the EWM with adding abnormal data. To show the fluctuation of the corresponding weights more clearly in different algorithms with abnormal data, we separately list the normal weights and the weights of adding abnormal data for each algorithm in Fig. 5. From Fig. 5(a), the great weight fluctuations of indexes 1 and 2, 3 and 4, 7 and 8 were caused by the abnormal data added, and the weight stability of the overall indexes has changed. Figure 5(b) shows that in the method i-AHP, abnormal data caused fluctuations in the weights of indexes 1 and 2, 3 and 4, 7 and 8. Regarding the WGMM method, the corresponding indexes 1 and 2, 7 and 8 also have unstable fluctuations in Fig. 5(c). It can be seen from Fig. 5(d) that the weights given by the proposed scheme avoid the problem of large weight fluctuation caused by the error of abnormal data. Thus, The proposed scheme outperforms other schemes, showing a more stable weight allocation.

**Figure 6:**
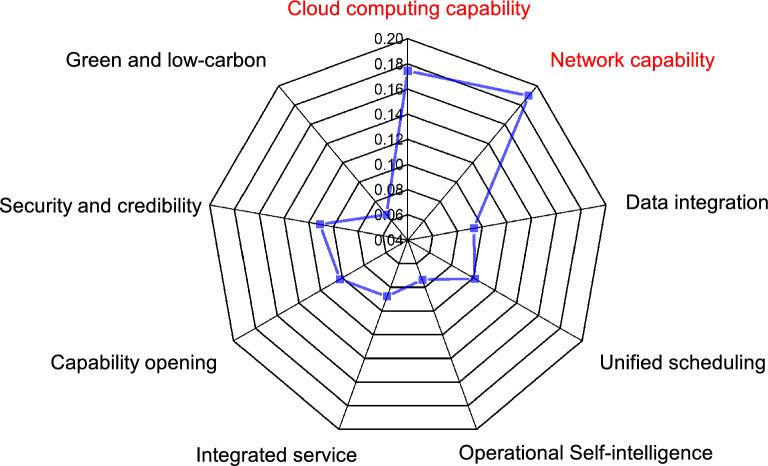
Index weight values corresponding to different algorithms for adding abnormal data.

Finally, according to the proposed scheme, we give the radar chart of 9-dimensional indexes of cloud-network convergence in Fig. 6. Through the radar chart, the importance of each index in the development of cloud-network convergence can be more clearly reflected. Compared with other indexes, we find that cloud computing capability and network capability play an important role in the development of cloud-network convergence. We provide a reference for the future development direction of cloud-network convergence.

### Remark 2

The most important steps of evaluation in actual operation are index scoring and index weight assignment. Our scheme mainly focuses on index weight. The reference methods of obtaining index weight, AHP and EWM, both need data, one is subjective data and the other is objective data. The difficulty lies in that subjective data needs professional scoring by experts in the field of cloud-network convergence, and objective data needs to be filled in by experts in the corresponding fields of each index according to the actual situation. In order to verify the effectiveness of the scheme, we have selected the authoritative cloud-network convergence experts and experts in the corresponding fields of various indicators to supplement the data. However, the authority of experts cannot be determined. Therefore, the final simulation results are only used to verify the effectiveness of the proposed scheme, and the actual application needs the data of more authoritative experts.

## Conclusion

Leveraging AHP and the EWM, this paper proposes an index evolution system, including index selection scheme and index evaluation scheme. Our important conclusion is that an evaluable evaluation index for cloud-network convergence is proposed based on the current development status of cloud-network convergence. To quantify the influence of each index on the development of cloud-network convergence, we design i-AHP index selection and stable comprehensive index weight scheme for cloud-network convergence. In addition, we add random abnormal data that meet the index scoring rules to verify the stability of the proposed scheme. Compared with the common EWM, i-AHP, and other methods, the proposed scheme has better stability in index weight evaluation.

In addition, with the development of cloud-network convergence in the future, the index’s type and scoring rules will change correspondingly, but our proposed scheme is still available. However, it is inevitable that a new evaluation scheme will appear, which is more suitable for evaluating the capability of cloud-network convergence. Generally, a single evaluation method has its shortcomings, and the advantages of various evaluation methods can be comprehensively considered in future research. It is suggested that based on the characteristics of the evaluated object, the idea of this paper and related methods in CDMA field should be comprehensively analyzed, and a more applicable and stable scheme should be put forward.

## Data Availability

All data generated or analysed during this study are included in this published article.

## Appendix

In the appendix, we explain the technical vocabulary involved in this paper as follows:

### Cloud-network convergence

It is a profound change of information infrastructure by the integration of communication and information technologies. The independent cloud computing resources and network facilities are merged to form a system of integrated supply, integrated operation and integrated service

### Network interface card

It is a circuit board installed in a computer and can provide a private network connection with the computer

### SR-IOV

Single root I/O virtualization allows devices (such as network adapters) to separate access to their resources among various PCIe hardware functions

### DPDK

It is the data plane development kit that consists of libraries to accelerate packet processing workloads running on a wide variety of CPU architectures

### Network slicing

It enables you to build multiple logical networks, i.e., network slices, on top of a common shared physical infrastructure
